# Changes in Access to Health Services of the Immigrant and Native-Born Population in Spain in the Context of Economic Crisis †

**DOI:** 10.3390/ijerph111010182

**Published:** 2014-09-30

**Authors:** Irene Garcia-Subirats, Ingrid Vargas, Belén Sanz-Barbero, Davide Malmusi, Elena Ronda, Mónica Ballesta, María Luisa Vázquez

**Affiliations:** 1 Health Policy and Health Services Research Group, Health Policy Research Unit, Consorci de Salut i Social de Catalunya, Avenida Tibidabo, 21, Barcelona 08022, Spain; E-Mails: ivargas@consorci.org (I.V.); mlvazquez@consorci.org (M.L.V.); 2 Sub-Program on Immigration and Health of the CIBERESP, Melchor Fernández Almagro, 3–5, Madrid 28029, Spain; E-Mails: dmalmusi@aspb.cat (D.M.); elena.ronda@ua.es (E.R.); monica.ballesta@carm.es (M.B.); 3 National School of Public Health, Instituto de Salud Carlos III, Avda Monforte de Lemos 5, Pabellón 7, Madrid 28029, Spain; E-Mail: bsanz@isciii.es; 4 CIBERESP, Melchor Fernández Almagro, 3–5, Madrid 28029, Spain; 5 Agència de Salut Pública de Barcelona (IIB-Sant Pau), Plaça Lesseps 1, Barcelona 08023, Spain; 6 Public Health Department, University of Alicante, Carretera de San Vicente del Raspeig, s/n, Alicante 03690, Spain; 7 Center for Research in Occupational Health (CISAL), Universitat Pompeu Fabra, Doctor Aiguader, 88, Barcelona 08003, Spain; 8 Department of Epidemiology, Consejería de Sanidad y Política Social de la Región de Murcia, Murcia Regional Heath Authority, Ronda Levante, 11, Murcia 30008, Spain

**Keywords:** immigration, health care utilization, access to health care, economic crisis, Spain

## Abstract

Aim: To analyze changes in access to health care and its determinants in the immigrant and native-born populations in Spain, before and during the economic crisis. Methods: Comparative analysis of two iterations of the Spanish National Health Survey (2006 and 2012). Outcome variables were: unmet need and use of different healthcare levels; explanatory variables: need, predisposing and enabling factors. Multivariate models were performed (1) to compare outcome variables in each group between years, (2) to compare outcome variables between both groups within each year, and (3) to determine the factors associated with health service use for each group and year. Results: unmet healthcare needs decreased in 2012 compared to 2006; the use of health services remained constant, with some changes worth highlighting, such as the decline in general practitioner visits among autochthons and a narrowed gap in specialist visits between the two populations. The factors associated with health service use in 2006 remained constant in 2012. Conclusion: Access to healthcare did not worsen, possibly due to the fact that, until 2012, the national health system may have cushioned the deterioration of social determinants as a consequence of the financial crisis. Further studies are necessary to evaluate the effects of health policy responses to the crisis after 2012.

## 1. Introduction

In 2011, Spain’s immigrant population declined slightly for the first time since the beginning of the migratory surge that characterized the first decade of the 21st century [1]. Nevertheless, Spain remains one of the European countries with the highest volume of foreigners, at 11.7% of the population in 2013 [2]. The sociodemographic change that immigration has brought about in the country is reflected in the significant number of recent publications that analyze different aspects of the immigrant population, including healthcare access and health service use [3,4,5,6,7,8,9]. Many of these studies compare the use of services between the immigrant and autochthonous populations [3,4,5,6,7,8,9]. Reviews of national and European literature [10,11,12] show that, in general, given the same health needs, use of primary care services is similar among the immigrant population from low-income countries and the autochthonous population. However, this immigrant population has lower use of specialist care and higher use of emergency services. Explaining these differences requires a specific analysis of the determinants of health services use, which is a topic that has been scarcely addressed in national [8,13] and European [14] literature. At the international level, no studies were found that show the specific determinants for each group, although one study shows that differences in health service use between the two groups is due to the relatively worse social and economic situation of immigrants [14]. Only one study in Spain compares the determinants of health service use between the immigrant and autochthonous populations and it concludes that there are similar socioeconomic determinants related to health service use in both populations [8]. The study shows that women make more use of specialist and hospitalization services, that students make less use of primary care and specialist services than do employees, and that those with poor health make more use of health services at all levels of care. The study also shows that holding a private health insurance policy is associated with lower use of primary care and greater use of specialist care [8]. In contrast, other factors, such as education level and income, are associated with the use of health services in the autochthonous population but not in the immigrant population.

Prolonged economic crises, such as that experienced in Spain since 2008, bring about an increase in the number of citizens experiencing social and economic vulnerability [15]; these individuals are more susceptible to the effects of the crisis, which could include a general worsening of health, a decrease in access to health services [16] or an increase in barriers to access to healthcare [17]. The immigrant population, along with elderly and those with few economic resources (due to unemployment or low incomes) are among the most fragile social groups and can suffer more negative consequences as a result of the economic crisis. At the international level, the study by Lusardi *et al.* [18] shows a decrease in health service use as a consequence of the crisis in five countries, particularly for households in economic distress. However, the results differ according to the health system of each country; those countries with universal health systems and lower copayments experience a smaller decline in health service use. Another study carried out in eight European countries affected by the crisis, including Spain, shows a general increase in unmet health needs (measured as the percentage of people that needed medical consultation or treatment but did not receive it) during the crisis. This increase was related to unmet need due to wait times, cost and transportation—although behavior is different for each country [19]. Until recently, studies carried out in Spain on the effects of the economic crisis have focused on its impact on population health [20,21]. Only one study analyzes the effects of the crisis on the general population’s access to health services, showing a decrease in health service use compared to 2006, especially among unskilled workers [22].

The Spanish national health system, which was characterized by universal access until 2012 in addition to specific policies designed to improve access to care for the immigrant population [23,24], may have cushioned the adverse consequences of the crisis during its first years. Analysis of the most recent Spanish National Health Survey [25], carried out between 2011 and 2012, four years after the beginning of the economic crisis, permits a first approach to the impact of the crisis on health access and health service use [26].

One of the most frequently used theoretical frameworks to analyze access to health services is that of Aday and Andersen [27]. This framework distinguishes between actual access or use of services, and potential access or analysis of determinants, differentiating between individual factors (predisposing, enabling and need) and health service factors. Another approach that is increasingly used [28,29] in the analysis of access is the measurement of unmet health needs, that is, the persistence of need due to lack of adequate care [28]. Analyzing unmet need permits identifying barriers to access throughout the continuum of care, given the same health need. These two approaches oriented this study. The objective is to analyze the changes in access to health care and the determinants of access among the immigrant and autochthonous populations in Spain between 2006 and 2012.

## 2. Method

### 2.1. Design

A comparative analysis of two cross-sectional studies in Spain was carried out; the Spanish National Health Survey (SNHS) of 2006/07 [30] and the SNHS of 2011/12 [31]. Both were conducted with representative samples of the non-institutionalized Spanish population.

### 2.2. Study Population and Sample

This study considered the population born in low and middle-income countries living in Spain and the Spanish-born population. The foreign-born population from high-income countries, according to the International Monetary Fund classification [32], was excluded since these immigrants are not particularly disadvantaged in terms of living conditions and health status [33]. Only the population of 16 to 59 years of age was included to ensure that both population groups would be comparable in terms of age. For both years, the percentage of those over age 60 born in low and middle-income countries is less than 5%, while over 25% of the autochthonous population is over 60. This restricts the sample to 21,818 people in the SNHS 2006/07 and 15,200 in the SNHS 2011/12, with a proportion of immigrants of 13.3% in 2006 and 15.9% in 2012.

Three-stage stratified sampling was conducted in both surveys. In the first stage, census tracts were selected, stratified by the size of the municipality. Within each stratum, census sections were selected with a probability proportional to their size. In the second stage, households were selected with equal probability within each section by systematic sampling from a list with random start. Finally, an adult (from the list of survey eligible persons in the household at the time the interview was carried out) was randomly selected (aged 16 or over in the SNHS 2006/07 and 15 or over in the SNHS 2011/12) to fill out the Adult Questionnaire [34,35]. Information was collected between June of 2006 and June of 2007 (SNHS 2006/07), and from July of 2011 to June of 2012 (SNHS 2011/12) through personal interviews.

### 2.3. Variables

Outcome variables were: unmet healthcare need in the last 12 months defined as the lack of health care when needed and the reason for not receiving it. Unmet healthcare need was assessed through the question “In the last 12 months, did you ever need medical care and not receive it?” and the reason for not receiving care was assessed through the question “What was the main reason why you did not receive such assistance?” The other outcome variables were the use of different levels of care (yes; no) (both public and private): primary care (visit to a general practitioner in the 4 weeks before the interview), specialized care (visit to a specialist in the 4 weeks before the interview), hospitalization (in the past year) and emergency services visits (in the past year). Hospitalization and emergency services visits for childbirth were excluded.

Immigrants were defined in this study as people born in low and middle-income countries as classified by the International Monetary Fund.

Following the Aday and Andersen model the explanatory variables included were: (a) need factors: *self-rated health* assessed through the question, “Within the last twelve months, would you say your health was very good, good, fair, poor or very poor”; results were categorized as good (good or very good) and poor (fair, poor or very poor); *suffering from a chronic disease* (self-declared) (none, one, two, three or more) and *having suffered an injury* in the past year (yes; no); (b) predisposing factors: *sex* (man; woman), *age* (16 to 29; 30 to 44; 45 to 59); and (c) enabling factors: *holding a private health insurance policy* (yes; no), *employment situation* (paid worker; unemployed; others that include: students, domestic workers, voluntary workers, pensioners, retired, disabled), *civil status* (married or single, separated, divorced and widowed) and *social class* based on the person’s current or last occupation. Those surveyed who had never had an occupation were classified according to the social class of the head of household. Following the Spanish Society of Epidemiology classification [36,37], social class was regrouped into four categories: (i) class I-II: higher-level professionals, administrative managers, directors of large companies, medium-level professionals and directors of small companies; (ii) class III: administrative workers, clerks, safety and security workers and self-employed workers, (iii) class IV: skilled and semi-skilled manual occupations; (iv) class V: unskilled manual occupations. To describe the study sample, two more variables were analyzed: *education level* (no studies; primary; secondary; university) and *years of residence in Spain* for immigrants in the SNHS 2011/12 (less than 5 years; from 5 to 10; more than 10).

### 2.4. Statistical Analysis

The databases were merged and the variable “year of interview” was created (2006 and 2012). First, a descriptive analysis of the dependent variables was carried out by year and origin (autochthons and immigrants). Second, the percentage of unmet need was calculated for immigrants and autochthons, and a logistic regression model was used to analyze whether there had been changes in 2012 with respect to 2006 for each group, and whether the difference between groups had changed, adjusted by the explanatory variables. Third, health service use was analyzed. Poisson regression models were estimated with robust variance, using the prevalence ratio (PR) at the 95% confidence interval (CI 95%) to (a) compare the changes in the prevalence of health services use in 2012 compared to 2006 for immigrants and autochthons, adjusted by the explanatory variables; (b) compare the prevalence of health service use of the immigrant population with respect to the autochthonous population for each year; and (c) determine the factors associated with health service use for each year and each level of care.

All of the analysis included weights derived from the complex sample design. Statistical software SPSS 17 and STATA 12 were used.

## 3. Results

### 3.1. Sociodemographic Characteristics of the Sample

For both years, women made up half of the autochthonous population, while the proportion of women in the immigrant population was slightly higher. In both groups, the percentage of young people (aged 16 to 29) decreased, and the percentage of adults (aged 45 to 59) increased with respect to 2006. The percentage of adults was greater in the autochthonous population (35.6%) than in the immigrant population (19.9%). The percentage of people with university studies increased in both populations, although more so for autochthons. There were few recently arrived immigrants in the country; the vast majority (83.0%) had resided more than five years in Spain. Fifty-two percent was from Latin America, followed by Eastern Europe (22.7%) and the Maghreb countries (14.2%). The employment situation in Spain changed notably during these five years. The percentage of people unemployed increased from 9.1% to 17.9% for autochthons and from 10.3% to 26.9% for immigrants. The percentage of people with private health insurance did not vary in the autochthonous population but decreased in the immigrant population. The perception of good health increased in 2012 with respect to 2006 in both groups. About half of immigrants (50.4%) declared no chronic disease compared to 37.8% of autochthons. The percentage of people who had had an injury in the past year decreased in both groups (Table 1).

**Table 1:** Sociodemographic characteristics and health needs in autochthons and immigrants. SNHS 2006/07 and 2011/12.

	Autochthons		Immigrants from low-income countries	
	2006	2012	2006	2012
	n (%)	n (%)	n (%)	n (%)
**Sociodemographic characteristics**				
**Sex**				
*Men*	9470 (51.2)	6403 (51.0)	1370 (47.4)	1150 (48.1)
*Women*	9034 (48.8)	6156 (49.0)	1523 (52.6)	1240 (51.9)
**Age**				
*16–29*	5137 (27.8)	3153 (25.1)	1180 (40.8)	761 (31.8)
*30–44*	7426 (40.1)	4937 (39.3)	1242 (42.9)	1154 (48.3)
*45–59*	5941 (32.1)	4470 (35.6)	472 (16.3)	476 (19.9)
**Education level**				
*No studies*	714 (3.9)	398 (3.2)	184 (6.4)	144 (6.0)
*Primary*	9091 (49.6)	5458 (43.5)	1213 (42.2)	1019 (42.6)
*Secondary*	4699 (25.6)	3131 (24.9)	1057 (36.8)	817 (34.2)
*University*	3827 (20.9)	3572 (28.4)	420 (14.6)	410 (17.2)
**Time of residence in Spain**				
*0–4 years*				405 (17.1)
*5–10 years*				1309 (55.2)
*More than 10 years*				659 (27.8)
**Country of birth**				
*Europe*			587 (20.3)	543 (22.7)
*Latin America*			1633 (56.4)	1243 (52.0)
*Maghreb*			459 (15.9)	340 (14.2)
*Sub-Saharan Africa*			114 (3.9)	110 (4.6)
*Asia*			101 (3.5)	154 (6.4)
**Social class**				
*I–II (directors and managers)*	4198 (23.1)	2523 (20.5)	293 (10.3)	121 (5.2)
*II*	4668 (25.7)	2703 (22.0)	288 (10.2)	223 (9.6)
*III*	7314 (40.2)	5238 (42.6)	1498 (52.8)	1156 (49.6)
*IV (unskilled workers)*	2015 (11.1)	1830 (14.9)	756 (26.7)	832 (35.7)
**Private health insurance**				
*Yes*	3115 (16.8)	1999 (15.9)	261 (9.3)	152 (6.4)
**Employment situation**				
*Paid work*	12105 (65.5)	7457 (59.5)	2095 (72.5)	1220 (51.2)
*Unemployed*	1683 (9.1)	2247 (17.9)	297 (10.3)	641 (26.9)
*Other ^a^*	4689 (25.4)	2837 (22.6)	496 (17.2)	521 (21.9)
**Civil status**				
*Married*	8359 (45.3)	5955 (47.4)	1415 (48.9)	1115 (46.7)
*Single. separated or divorced. widowed*	10112 (54.7)	6596 (52.6)	1476 (51.1)	1272 (53.3)
**Need**				
**Perceived health**				
*Good (very good or good)*	13,962 (75.5)	10,147 (80.8)	2073 (71.7)	1863 (77.9)
*Poor (regular. poor or very poor)*	4541 (24.5)	2412 (19.2)	820 (28.3)	528 (22.1)
**Chronic disease**				
*None*	5488 (29.7)	4743 (37.8)	1138 (39.3)	1205 (50.4)
*One*	4123 (22.3)	2933 (23.4)	634 (21.9)	553 (23.1)
*Two*	2951 (15.9)	1780 (14.2)	415 (14.3)	237 (9.9)
*Three or more*	5941 (32.1)	3103 (24.7)	705 (24.4)	396 (16.6)
**Injury**				
*Yes*	1971 (10.7)	1084 (8..6)	294 (10.2)	186 (7.8)

Note: *^a^*: Other situations include: students, domestic workers, retired, pensioners, disabled.

### 3.2. Unmet Health Needs

**Figure 1:**
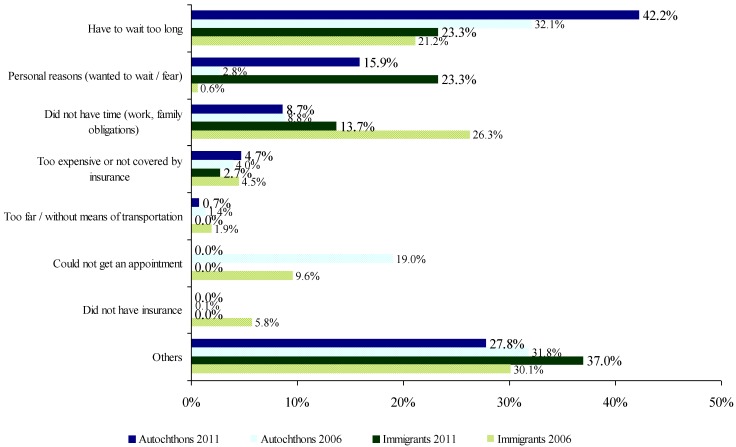
Reasons for not receiving health care services. SNHS 2006/07 and 2011/12.

Unmet need for healthcare decreased significantly in 2012 with respect to 2006 in both autochthons (PR: 0.61%, CI 95%: 0.52–0.72) and immigrants (PR: 0.65, CI 95%: 0.44–0.98%) (Table 2). The principal reason for unmet need in 2012 was related to health services characteristics: both groups referred to long wait times, which increased in 2012, especially for autochthons (42.2%) (Figure 1). It is worth noting that the high percentage of immigrants who referred to the lack of time due to work in 2006 (26.3%) decreased considerably in 2012 (13.7%) (this answer option was separated from the option “lack of time due to family duties” in 2006). In both groups, “waiting to see whether the problem resolved itself” increased compared to 2006 particularly for the immigrant population. It is also worth mentioning that not all of the reasons behind unmet needs were comparable, because the answer options for each year were different.

### 3.3. Use of Health Services by Level of Care

After adjusting for need and the other explanatory variables, the use of health services for both autochthons and immigrants did not change significantly in 2012 with respect to 2006, with the exception of general practitioner visits for autochthons, which decreased (PR_2012_: 0.91, CI: 0.87–0.96) (Table 2).

No significant differences were found between both populations for any level of care in 2012. However, adjusting for need and the other explanatory variables revealed some relevant results that are worth noting. In 2012 the immigrant population had a higher prevalence of visiting the general practitioner (24.5%) than the autochthonous population (22.5%) compared to 2006. The difference in the use of specialist visits (14.3% for autochthons and 10.7% for immigrants in 2006) was similar in 2012 (14.1% for autochthons and 11.0% for immigrants), although not significant (PR_Inmigrants_: 0.84, CI: 0.70–1.02). For both populations hospital visits (excluding births) in the past year were around 6%. The use of emergency services (also excluding births) decreased slightly for both groups, and the difference between them, while not statistically significant, was maintained from 2006 to 2012; the autochthonous population showed a lower prevalence of use of this care level (26.2% compared to 30.4% of the immigrant population) (Table 2).

**Table 2:** Changes in unmet health need and in the use of different levels of care for autochthons and immigrants by year and group.

	Autochthons			Immigrants			Immigrants Compared to Autochthons	
	2006	2012	PR ^a^ (IC 95%)	2006	2012	PR ^a^ (IC 95%)	2006	2012
	n (prev)	n (prev)		n (prev)	n (prev)		PR ^b^ (IC 95%)	PR ^b^ (IC 95%)
**Unmet need**	731 (4.0)	277 (2.2)	**0.61 (0.52–0.72)**	162 (5.6)	73 (3.1)	**0.65 (0.44–0.98)**	1.21 (0.89–1.63)	1.22 (0.86–1.74)
**Health service use**								
**Visit to general practitioner**	4696 (25.5)	2821 (22.5)	**0.91 (0.87–0.96)**	683 (23.9)	586 (24.5)	1.02 (0.86–1.21)	0.91 (0.80–1.03)	1.01 (0.89–1.15)
**Visit to specialist**	2619 (14.3)	1773 (14.1)	1.08 (1.01–1.16)	304 (10.7)	262 (11.0)	1.03 (0.79–1.34)	**0.76 (0.63–0.91)**	0.84 (0.70–1.02)
**Hospitalization (not births) ^c^**	1149 (6.2)	700 (5.6)	1.02 (0.91–1.15)	174 (6.0)	141 (5.9)	1.07 (0.73–1.58)	0.99 (0.74–1.33)	1.06 (0.80–1.41)
**Emergency (not births) ^c^**	5242 (28.3)	3290 (26.2)	1.02 (0.98–1.07)	941 (32.5)	728 (30.4)	1.04 (0.90–1.18)	1.06 (0.96–1.17)	1.06 (0.95–1.18)

Notes: **Prev**: Prevalence; **PR** ^a^: Prevalence ratio to compare the use of health services between 2006 and 2012 in each population group adjusted by sex, age, social class, private health insurance, employment situation, civil status and perceived health; **PR** ^b^: Prevalence ratio to compare the use of health services between autochthons and immigrants in each year adjusted by sex, age, social class, private health insurance, employment situation, civil status and perceived health; ^c^: In the models of hospital and emergency service use values are also adjusted by having suffered an injury in the past year.

### 3.4. Factors Associated with the Use of Different Levels of Care

The determinants of health service use were analyzed according to the Aday and Andersen conceptual framework (Table 3, Table 4, Table 5 and Table 6). No relevant changes in the determinants of the groups’ health service use between the two years were found, although we observed some differences between both population groups related to levels of care. With respect to need, the perception of poor health and suffering from a chronic disease were common factors in both groups and were associated with greater use of any level of care, for both years. For hospitalizations and use of emergency services, having suffered an injury in the past year was significantly associated with greater use of services in both groups for both years.

In terms of visits to the general practitioner (Table 3), women made greater use of this level of care (significantly in autochthons, and in immigrants in 2012), in both years. In the autochthonous population, people from lower social classes were more likely to use general practitioner services in both years (significantly). In contrast, there were no differences by social class for the immigrant population. Having private health insurance was associated with decreased use in both groups, but it was only significant for autochthons in 2006.

**Table 3:** Determinants of general practitioner visits in autochthons and immigrants in 2006 and 2012.

	Autochthons						Immigrants					
	2006		2012		2006		2012	
	Prev	PR (CI 95%)	Prev	PR (CI 95%)	Prev	PR (CI 95%)	Prev	PR (CI 95%)
**Predisposing factors**								
Sex								
*Men*	21.1	1	18.9	1	19.3	1	17.9	1
*Women*	30.1	**1.18 (1.10–1.27)**	26.2	**1.22 (1.13–1.33)**	27.9	1.20 (0.91–1.56)	30.6	**1.43 (1.09–1.87)**
Age								
*16–29*	21.5	1	19.2	1	21.9	1	20.1	1
*30–44*	23.3	0.90 (0.81–1.01)	20.9	1.02 (0.89–1.16)	22.6	0.90 (0.68–1.18)	25.5	0.99 (0.73–1.34)
*45–59*	31.7	0.93 (0.83–1.04)	26.6	1.01 (0.89–1.15)	32.0	1.14 (0.82–1.59)	29.2	0.97 (0.69–1.36)
**Enabling factors**								
Social class								
*I–II (directors and managers)*	20.5	1	17.4	1	23.0	1	30.8	1
*II*	24.9	**1.11 (1.01–1.23)**	20.7	1.11 (0.97–1.27)	21.9	0.83 (0.50–1.39)	21.1	0.67 (0.39–1.13)
*III*	27.8	**1.14 (1.04–1.26)**	23.3	**1.19 (1.06–1.34)**	24.6	1.07 (0.76–1.51)	21.7	0.71 (0.47–1.08)
*IV (unskilled workers)*	29.2	**1.16 (1.03–1.32)**	28.9	**1.24 (1.09–1.43)**	23.6	1.11 (0.75–1.63)	28.1	0.79 (0.52–1.22)
Private health insurance								
*No*	26.6	1	23.1	1	25.0	1	24.9	1
*Yes*	20.2	**0.85 (0.77–0.94)**	19.0	0.92 (0.82–1.04)	19.3	0.80 (0.55–1.16)	18.4	0.80 (0.51–1.25)
Employment situation								
*Workers*	23.5	1	19.9	1	21.9	1	24.8	1
*Unemployed*	29.1	1.05 (0.93–1.18)	24.5	1.08 (0.97–1.19)	27.1	1.12 (0.77–1.62)	22.2	0.96 (0.72–1.27)
*Other ^a^*	29.4	1.04 (0.96–1.12)	27.6	**1.14 (1.04–1.26)**	30.2	1.21 (0.90–1.63)	26.5	1.15 (0.85–1.56)
Civil status								
*Married*	28.0	1	23.1	1	26.7	1	26.4	1
*Single. separated. divorced or widowed*	22.5	0.93 (0.86–1.01)	21.8	1.05 (0.97–1.14)	21.0	0.86 (0.67–1.09)	22.0	0.84 (0.67–1.05)
**Need**								
Self-rated health								
*Good*	18.7	1	17.8	1	19.9	1	20.0	1
*Poor*	46.4	**1.85 (1.71–1.99)**	42.2	**1.79 (1.64–1.96)**	33.8	**1.32 (1.03–1.7)**	40.5	**1.37 (1.06–1.76)**
Chronic disease								
*None*	13.4	1	14.4	1	17.3	1	16.3	1
*One*	20.8	**1.44 (1.26–1.64)**	20.5	**1.37 (1.21–1.56)**	21.7	1.16 (0.81–1.67)	25.4	**1.55 (1.11–2.17)**
*Two*	26.2	**1.68 (1.46–1.94)**	22.7	**1.39 (1.21–1.59)**	27.6	1.39 (0.97–1.99)	35.4	**1.99 (1.41–2.81)**
*Three or more*	39.7	**2.05 (1.81–2.32)**	36.4	**1.80 (1.59–2.03)**	34.3	**1.55 (1.10–2.16)**	41.9	**2.07 (1.48–2.89)**

Notes: **Prev**, Prevalence; **PR**, Prevalence ratio adjusted by age, sex, social class, private health insurance, employment situation, civil status and perceived health; ^a^ Other situations include: students, domestic workers, retired, pensioners, disabled.

Regarding specialist visits (Table 4), women showed greater use in both groups, both in 2006 and 2012, compared to men. In 2006, social class was not significantly associated with use in autochthons, but it was significant in 2012; lower social classes were less likely to use this level of care (PR_CS III_: 0.76, CI 95%: 0.66–0.87 and PR_CS IV_: 0.66, CI 95% 0.64–0.91). The prevalence of service use by social class in immigrants showed a gradient of inequality, although not significantly, and the difference between the two extreme social classes was greater in 2012 than in 2006. Having private health insurance was significantly associated with greater use of specialist visits for both groups for both years.

**Table 4:** Determinants of specialist visits in autochthons and immigrants in 2006 and 2012.

	Autochthons				Immigrants			
	2006		2012		2006		2012	
	Prev	PR (CI 95%)	Prev	PR (CI 95%)	Prev	PR (CI 95%)	Prev	PR (CI 95%)
**Predisposing factors**								
Sex								
*Men*	10.9	1	11.1	1	6.2	1	6.2	1
*Women*	17.9	**1.38 (1.241.53)**	17.3	**1.32 (1.18–1.47)**	14.6	**2.08 (1.29–3.36)**	15.4	**2.24 (1.46–3.46)**
Age								
*16–29*	10.7	1	10.1	1	8.4	1	7.6	1
*30–44*	14.6	1.06 (0.9–1.25)	13.5	1.01 (0.85–1.21)	10.9	1.11 (0.70–1.76)	12.0	1.46 (0.94–2.27)
*45–59*	17.2	0.95 (0.8–1.13)	17.7	1.00 (0.84–1.20)	15.6	1.27 (0.73–2.19)	13.9	1.45 (0.87–2.43)
**Enabling factors**								
Social class								
*I–II (directors and managers)*	*13.7*	*1*	*15.5*	*1*	*12.5*	*1*	*15.0*	*1*
*II*	14.9	1.04 (0.92–1.19)	15.9	0.96 (0.83–1.11)	16.7	1.36 (0.74–2.50)	12.1	0.84 (0.39–1.81)
*III*	14.2	0.94 (0.83–1.07)	12.3	**0.76 (0.66–0.87)**	8.8	0.85 (0.52–1.41)	10.6	0.85 (0.43–1.68)
*IV (unskilled workers)*	14.5	0.94 (0.79–1.11)	15.1	**0.76 (0.64–0.91)**	11.6	1.25 (0.71–2.21)	10.3	0.65 (0.32–1.35)
Private health insurance								
*No*	13.7	1	12.8	1	9.9	1	10.5	1
*Yes*	17.4	**1.34 (1.19–1.50)**	20.9	**1.62 (1.43–1.83)**	18.4	**1.83 (1.22–2.75)**	18.4	**1.92 (1.14–3.23)**
Employment situation								
*Workers*	13.5	1	13.5	1	9.3	1	11.1	1
*Unemployed*	15.1	0.99 (0.84–1.16)	13.4	0.98 (0.85–1.14)	17.1	1.61 (0.99–2.62)	10.9	1.17 (0.79–1.74)
*Other ^a^*	16.2	1.03 (0.92–1.15)	16.4	1.13 (0.99–1.28)	12.2	1.01 (0.63–1.62)	10.7	1.12 (0.71–1.78)
Civil status								
*Married*	16.0	1	16.0	1	8.8	1	9.9	1
*Single. separated. divorced or widowed*	12.3	0.99 (0.88–1.11)	12.1	0.91 (0.82–1.02)	12.5	0.85 (0.58–1.23)	12.2	1.37 (0.97–1.93)
**Need**								
Self-rated health								
*Good*	9.8	1	9.9	1	7.9	1	8.4	1
*Poor*	28.6	**2.19 (1.97–2.43)**	31.7	**2.54 (2.26–2.85)**	17.6	**1.62 (1.02–2.57)**	20.1	**1.67 (1.15–2.43)**
Chronic disease								
*None*	6.2	1	7.3	1	7.4	1	7.8	1
*One*	11.3	**1.60 (1.33–1.92)**	13.2	**1.61 (1.36–1.90)**	6.7	0.80 (0.45–1.42)	10.9	1.20 (0.74–1.94)
*Two*	14.9	**1.95 (1.60–2.37)**	14.8	**1.54 (1.27–1.87)**	13.0	1.24 (0.72–2.14)	13.1	1.33 (0.74–2.40)
*Three or more*	23.8	**2.41 (2.03–2.86)**	25.0	**2.05 (1.74–2.43)**	18.3	1.34 (0.68–2.67)	19.4	1.41 (0.87–2.30)

Notes: **Prev**, Prevalence; **PR**, Prevalence ratio adjusted by age, sex, social class, private health insurance, employment situation, civil status and perceived health; ^a^ Other situations include: students, domestic workers, retired, pensioners, disabled.

With respect to hospitalizations (Table 5), autochthonous women showed significantly less hospitalization than men for both years. Although the prevalence of hospitalizations was higher in the poorer social classes, when need for healthcare was taken into account, the association between social class and hospitalizations was not significant. Having private health insurance was associated with greater use of hospitalizations in both years for the autochthonous population (PR_2012_: 1.63, CI 95%: 1.33–2.00), but not for immigrants.

**Table 5:** Determinants of hospitalization in autochthons and immigrants in 2006 and 2012.

	Autochthons				Immigrants			
	2006		2012		2006		2012	
	Prev	PR (CI 95%)	Prev	PR (CI 95%)	Prev	PR (CI 95%)	Prev	PR (CI 95%)
**Predisposing factors**								
Sex								
*Men*	6.6	1	5.6	1	5.8	1	6.2	1
*Women*	5.8	**0.74 (0.63–0.87)**	5.5	**0.83 (0.70–0.99)**	6.2	1.00 (0.52–1.91)	5.7	0.77 (0.43–1.37)
Age								
*16–29*	4.8	1	4.4	1	5.0	1	4.9	1
*30–44*	5.5	1.04 (0.81–1.35)	5.1	0.98 (0.73–1.30)	7.0	1.11 (0.58–2.09)	5.6	0.97 (0.52–1.81)
*45–59*	8.3	1.19 (0.92–1.55)	6.8	0.95 (0.71–1.27)	5.9	0.80 (0.32–2.03)	8.2	1.15 (0.56–2.37)
**Enabling factors**								
Social class								
*I–II (directors and managers)*	*5.5*	*1*	*4.4*	*1*	*3.8*	*1*	*4.1*	*1*
*II*	5.8	0.95 (0.76–1.19)	5.9	1.22 (0.93–1.60)	9.0	2.11 (0.64–6.94)	5.8	1.30 (0.21–7.85)
*III*	6.4	0.97 (0.79–1.19)	5.8	1.03 (0.79–1.33)	6.3	1.43 (0.53–3.86)	5.7	1.34 (0.26–7.00)
*IV (unskilled workers)*	7.5	1.02 (0.77–1.36)	6.3	0.98 (0.73–1.31)	5.4	1.18 (0.38–3.62)	6.7	1.56 (0.29–8.58)
Private health insurance								
*No*	6.0	1	5.2	1	6.3	1	3.9	1
*Yes*	7.3	**1.41 (1.16–1.73)**	7.6	**1.63 (1.33–2.00)**	4.6	0.65 (0.25–1.70)	6.0	0.58 (0.19–1.77)
Employment situation								
*Workers*	5.7	1	4.8	1	6.3	1	6.3	1
*Unemployed*	7.6	1.18 (0.90–1.54)	6.5	1.21 (0.97–1.52)	6.1	0.91 (0.42–1.97)	6.2	0.93 (0.51–1.67)
*Other ^a^*	7.0	1.13 (0.93–1.36)	6.8	1.30 (1.05–1.61)	4.8	0.71 (0.35–1.42)	4.6	0.91 (0.40–2.07)
Civil status								
*Married*	6.4	1	5.7	1	6.2	1	6.7	1
*Single. separated. divorced or widowed*	5.9	1.11 (0.93–1.32)	5.4	1.02 (0.85–1.23)	5.9	1.02 (0.59–1.77)	5.0	0.83 (0.51–1.34)
**Need**								
Self-rated health								
*Good*	3.2	1	3.2	1	3.6	1	4.0	1
*Poor*	15.5	**4.43 (3.68–5.33)**	15.7	**4.25 (3.48–5.19)**	12.2	**2.72 (1.37–5.38)**	12.9	**2.33 (1.27–4.27)**
Chronic disease								
*None*	3.6	1	3.4	1	3.4	1	3.2	1
*One*	4.7	1.00 (0.75–1.33)	4.4	1.08 (0.82–1.44)	5.4	1.26 (0.56–2.84)	7.4	1.89 (0.87–4.07)
*Two*	5.9	1.10 (0.81–1.49)	5.4	1.09 (0.81–1.48)	6.7	1.23 (0.50–3.03)	6.8	1.58 (0.64–3.88)
*Three or more*	9.8	1.14 (0.87–1.50)	10.1	**1.41 (1.08–1.84)**	10.4	1.66 (0.65–4.25)	11.4	2.03 (0.96–4.28)
Injury								
*No*	5.5	1	4.9	1	5.6	1	5.0	1
*Yes*	11.8	**1.60 (1.31–1.96)**	12.8	**2.21 (1.76–2.76)**	9.9	**1.64 (0.71–3.80)**	16.7	**2.84 (1.50–5.37)**

Notes: **Prev**, Prevalence; **PR**, Prevalence ratio adjusted by age, sex, social class, private health insurance, employment situation, civil status and perceived health; ^a^ Other situations include: students, domestic workers, retired, pensioners, disabled.

Women in both groups had greater use of emergency services (Table 6) than men, although the relationship was significant only for the autochthonous population in 2012. The use of emergency services decreased with age for both groups and in both years, but it was only statistically significant for autochthons in 2012 (PR_30–44_: 0.85, CI 95%: 0.77–0.94 and PR_45–60_: 0.59, CI 95% 0.53–0.66). Social class was not significantly associated with use of this level of care for either group, although for autochthons, higher social class was related to lower prevalence of emergency service use. In immigrants, paid workers were more likely to use these services than those who were unemployed (PR: 0.75, CI 95%: 0.59–0.96).

**Table 6:** Determinants of the use of emergency services in autochthons and immigrants in 2006 and 2012.

	Autochthons				Immigrants			
	2006		2012		2006		2012	
	Prev	PR (CI 95%)	Prev	PR (CI 95%)	Prev	PR (CI 95%)	Prev	PR (CI 95%)
**Predisposing factors**								
Sex								
*Men*	27.5	1	24.3	1	31.5	1	28.4	1
*Women*	29.2	1.02 (0.96–1.09)	28.1	**1.11 (1.03–1.19)**	33.5	0.97 (0.79–1.19)	32.3	1.06 (0.86–1.31)
Age								
*16–29*	35.5	1	29.1	1	33.6	1	33.1	1
*30–44*	27.1	0.73 (0.67–0.79)	27.4	**0.85 (0.77–0.94)**	33.0	0.86 (0.71–1.05)	28.9	0.92 (0.72–1.18)
*45–59*	23.9	0.55 (0.51–0.60)	22.9	**0.59 (0.53–0.66)**	28.5	**0.64 (0.47–0.85)**	30.0	0.83 (0.62–1.11)
**Enabling factors**								
Social class								
*I–II (directors and managers)*	25.8	1	21.9	1	29.7	1	20.0	1
*II*	27.3	1.02 (0.94–1.12)	25.1	1.07 (0.96–1.20)	35.4	1.05 (0.69–1.57)	30.0	1.46 (0.81–2.65)
*III*	29.5	1.03 (0.95–1.12)	27.7	1.11 (1.00–1.22)	34.0	1.08 (0.78–1.50)	30.4	1.48 (0.88–2.49)
*IV (unskilled workers)*	30.7	1.04 (0.93–1.16)	29.5	1.11 (0.98–1.25)	29.1	0.93 (0.65–1.34)	31.9	1.48 (0.87–2.53)
Private health insurance								
*No*	28.4	1	26.1	1	32.7	1	30.3	1
*Yes*	27.9	1.03 (0.95–1.12)	26.8	1.06 (0.97–1.16)	34.1	0.91 (0.68–1.22)	32.9	1.01 (0.74–1.38)
Employment situation								
*Workers*	27.8	1	24.5	1	31.0	1	32.6	1
*Unemployed*	31.8	0.97 (0.88–1.08)	28.8	1.03 (0.94–1.13)	32.7	1.09 (0.82–1.45)	23.7	**0.75 (0.59–0.96)**
*Other ^a^*	28.4	0.95 (0.88–1.03)	28.3	1.01 (0.92–1.10)	38.8	1.23 (0.96–1.57)	33.6	1.05 (0.82–1.35)
Civil status								
*Married*	25.8	1	24.8	1	33.3	1	27.8	1
*Single. separated. divorced or widowed*	31.4	1.03 (0.96–1.10)	27.8	0.97 (0.9–1.05)	31.7	0.90 (0.74–1.08)	33.6	1.15 (0.94–1.40)
**Need**								
Self-rated health								
*Good*	23.0	1	21.5	1	26.6	1	48.9	1
*Poor*	44.8	**1.71 (1.6–1.83)**	46.1	**1.89 (1.74–2.04)**	47.6	**1.47 (1.21–1.78)**	25.2	**1.62 (1.31–2.01)**
Chronic disease								
*None*	21.1	1	19.8	1	25.3	1	24.5	1
*One*	25.0	1.15 (1.03–1.27)	23.9	1.20 (1.09–1.33)	33.0	1.23 (0.95–1.58)	33.7	1.27 (0.99–1.63)
*Two*	29.8	**1.35 (1.22–1.50)**	27.1	**1.29 (1.15–1.45)**	25.5	0.95 (0.69–1.29)	29.1	1.18 (0.84–1.64)
*Three or more*	36.6	**1.50 (1.37–1.65)**	37.5	**1.56 (1.41–1.72)**	47.9	**1.64 (1.29–2.08)**	44.8	**1.33 (1.02–1.72)**
Injury								
*No*	22.7	1	21.5	1	27.4	1	26.5	1
*Yes*	75.6	**2.87 (2.71–3.05)**	76.3	**3.24 (3.03–3.46)**	78.2	**2.84 (2.38–3.39)**	77.4	**2.69 (2.23–3.25)**

Notes: **Prev**, Prevalence; **PR**, Prevalence ratio adjusted by age, sex, social class, private health insurance, employment situation, civil status and perceived health; ^a^ Other situations include: students, domestic workers, retired, pensioners, disabled.

Finally, in the immigrant population, the effect of education level as a determinant of health service use was also analyzed in substitution for social class. The same results were found for the four levels of care, with no significant differences.

## 4. Discussion and Conclusions

This work represents a first approach to the study of changes in access to healthcare of the autochthonous and immigrant populations in Spain between 2006 and 2012. The two time periods existed within different socio-economic contexts: the first was a time of economic bonanza and the second was during the first years of a severe and sustained financial crisis. The results show that unmet healthcare need decreased in both groups in 2012 compared to 2006. Health service use remained similar, although there were some notable changes, such as the decrease in general practitioner visits for autochthons, the slight decrease in the use of emergency services by both groups, and a narrowed gap in specialist visits between both populations. The analysis of the determinants of access to healthcare shows that the factors associated with use of services in 2006 generally remained the same in 2012.

The decrease in unmet healthcare need, both in the autochthonous and immigrant population, could be associated with the increase in perception of good health during the same time period. These results agree with other studies [19,22], but do not agree with Eurostat data that points to an increase in unmet health need in Spain, particularly for the lowest income quintiles (2008–2012 period) [38]; this could be related to a different formulation of the question. The analysis of the reasons for unmet healthcare need show the presence of access barriers to care and changes during the time period, although these results should be interpreted with caution due to the difficulties in comparing the two surveys. They were explored through a close-ended question with contain different response options and because the category “*other reasons*” cannot be further analyzed, because there was no possibility to specify a different answer from the given list. On one hand, the increase in the lack of access to care due to wait times agrees with evidence regarding the increase in waiting times within the current healthcare context [22]. On the other hand, the marked decrease in the reason “*lack of time to attend health services due to work*” in the immigrant population could be related to the increase in unemployment. This was the primary reason for unmet need in the immigrant population in 2006 and was probably associated with the fear of losing a job [19,39] due to precarious working situations, also described in other studies [40].

The decrease in visits to a general practitioner in the autochthonous population, also described in another article [22], which reached a similar level to that of the immigrant population in 2012, could be due to the improvement in self-perceived health or a decrease in administrative visits. The slight decrease in the use of emergency services could be related to greater knowledge of the health system due to longer time of residence for the immigrant population [26]. For both immigrants and autochthons, it could also be related to the decrease in work related accidents possibly due to the increase in unemployment. Moreover, in 2012, unemployed immigrants made less use of emergency services than immigrant workers, a fact not observed in 2006 nor in existing literature [8]. This could be related to having the time necessary to visit a general practitioner. No differences were found in either population in terms of the use of emergency services in the two surveys, in contrast to the results of other studies [3,4,41]. This discrepancy could be due to the fact that the study population was under age 60, and in contrast, the other studies included the entire adult population. This would decrease the prevalence of emergency service use in autochthons such that it would seem to be less that than of the immigrant population.

In terms of specialist care, the significant difference between autochthons and immigrants in 2006, also described in other studies [3,4,41], did not appear in 2012. The difference could be due to immigrants’ less favorable social and economic situations [14] rather than their immigration status. Therefore, our results suggest that the National Health System that existed in Spain until 2012 tended towards diminishing inequalities in health service use among both populations, due to the universal access to health services in addition to specific policies developed to improve the health and access to care of the immigrant population [9,23]. However, it is important to note that equity in access to care does not guarantee equal access to high quality care. Inequalities related to access to quality care might still exist [9,23]. This is a subject that should be further studied and addressed by health policy.

The analysis of the determinants of health service use indicate that women make more use of both primary and specialist care services, which coincides with existing studies [8,13]. This is probably due to use related to maternity and gynecology services. The greater use of hospitalization [8] and emergency [13] services of immigrant women compared to autochthonous women that is described in other studies did not appear in this study, possibly because childbirth was excluded.

The most relevant difference found between both populations in this study was the pattern of inequity in the use of primary and specialist care, due to social class and the holding of a private health insurance policy on the part of the autochthonous population. Furthermore, this appears to have increased in 2012. Thus, the wealthier social classes make less use of primary care and greater use of specialist care services, corroborating the results of the only study that analyzes determinants separately [8]. It also agrees with those studies that analyze the determinants in the general population [42] including other socioeconomic indicators such as education level [4,41] or holding a private health insurance policy [8,43]. This inequality is less perceptible for the immigrant population, probably due to the lower proportion of immigrants in higher social classes, making it necessary to identify an indicator that better highlights the socioeconomic differences in this group.

Among the particularities of the determinants for the immigrant population, having a chronic disease was significant in determining visits to a general practitioner. This could be related to the length of residence in the country. The relatively good health immigrants compared to autochthonous population is lost over time as a consequence of adverse socioeconomic conditions; that is to say, immigrant health status declines and converges with the equivalent socioeconomic group in the autochthonous population [44].

One aspect worth noting concerning the immigrant population’s access to care is the fact that the SNHS does not permit the analysis of relevant factors that influence access to the National Health System such as administrative status, only analyzed in qualitative studies or in quantitative studies based on surveys specifically conducted with immigrants [9,45]. Administrative status has probably become an important barrier to access to care [46] due to the Royal Decree RD16/2012, which in addition to linking the right to health care to one’s affiliation to the Social Security, restricts the health rights of irregular immigrants to emergency and maternal healthcare and care for those under age 18 [47]. Given that the SNHS 2011/12 data collection took place before the decree, and that health budget reductions and other austerity policies in public spending were introduced after 2012 [48], further studies will be needed to evaluate their impact on access to care.

The principal limitation of this study is the lower participation of the immigrant population compared to the autochthonous population in the SNHS 2011/12. Despite the fact that the use of weighting factors gives this population as whole a weight similar to its assigned weight via the Municipal Register of Inhabitants [49], it is important to bear in mind that representation is different by country of origin. In addition, with the grouping of immigrants into a single population, there is a loss of heterogeneity. Some studies that disaggregate this population by continent of origin show differences in health service use [3,4,5,7,50]. As the aim of this study was to carry out a detailed analysis of the determinants of health service use, the sample size did not permit disaggregation at this level.

In conclusion, given existing data concerning access to care and health service use, access to health services did not get worse, rather we observed a decrease in unmet healthcare need and similar health service use among both groups, which could be attributed to a health system that, until 2012, maintained universal access, providing a cushion for the possible adverse effects of the crisis.
